# Iatrogenic Type A Dissection: Case Series and Surgical Management From a High-Volume Aortic Center

**DOI:** 10.1016/j.atssr.2023.02.011

**Published:** 2023-02-27

**Authors:** Amit Iyengar, John J. Kelly, Michael Catalano, Mark Helmers, William L. Patrick, Joshua Grimm, Joseph E. Bavaria, Nimesh D. Desai

**Affiliations:** 1 Division of Cardiovascular Surgery, Department of Surgery, University of Pennsylvania, Philadelphia, Pennsylvania; 2 Perelman School of Medicine, University of Pennsylvania, Philadelphia, Pennsylvania

## Abstract

**Background:**

Iatrogenic type A aortic dissection is a rare complication of surgical and nonsurgical cardiac procedures associated with high morbidity. The purpose of this study was to describe the intraoperative incidence, surgical management, and outcomes of iatrogenic type A dissections at our institution.

**Methods:**

Retrospective review of our institution’s adult cardiac surgery database was performed between 2002 and 2018 to identify all iatrogenic type A aortic dissection repairs. Operative reports were reviewed for cause of dissection and repair strategy. Follow-up surveillance for mortality and need for aortic reintervention was queried as available.

**Results:**

Overall, 36 patients undergoing iatrogenic type A repairs were identified (cardiac surgical incidence, 0.1%). Of these, 23 (63.9%) were related to open operation, 5 (13.9%) to percutaneous coronary interventions, 5 (13.9%) to thoracic endovascular repairs, and 3 (8.3%) to other endovascular procedures. Most patients underwent hemiarch repairs under circulatory arrest (28/36 [77.8%]), whereas total arch repair was required in 5 of 36 (13.9%). Among all patients, in-hospital mortality was 36.1% (13/36). Those who survived to discharge had low subsequent mortality, with no differences between endovascular and surgical causes (*P* = .797). On median follow-up of 3.1 years, need for redo aortic surgery was limited to 4 (11.1%) patients, all successfully treated with endovascular therapy.

**Conclusions:**

Iatrogenic type A dissections represent a rare but serious complication of cardiac procedures, with high in-hospital mortality for those undergoing surgical repair. A repair strategy involving an open distal anastomosis and proximal root reconstruction ensures durable freedom from need for redo surgery.

**In Short**

▪ Iatrogenic type A dissection is a rare but serious complication that carries mortality as high as 30% to 40%.
▪ Management should consist of prompt recognition and comprehensive repair with open distal anastomosis.

Iatrogenic type A aortic dissection stemming from cardiac surgical and interventional procedures is a rare but serious complication that carries a significant morbidity and mortality risk.^1^, ^2^, ^3^, ^4^, ^5^, ^6^, ^7^ Its incidence during cardiac surgery has been reported as 0.06% to 0.23% in previous studies with a 15% to 50% risk of mortality despite prompt recognition and intraoperative repair.^1^, ^2^, ^3^, ^4^ Various surgical repair strategies that take into consideration cannulation options, degree of cooling, assessment of root integrity, and origin and extent of the dissection have been proposed. In this manuscript, we sought to describe techniques and to evaluate outcomes of surgical repair for iatrogenic type A dissections at our high-volume aortic surgery center.

## Material and Methods

This study was approved by the institutional review board at the University of Pennsylvania. We performed a retrospective review of our adult cardiac surgery database from 2002 to 2018 to identify all adult patients undergoing iatrogenic type A aortic dissection repair. Follow-up was supplemented by review of the electronic medical record in January 2022.

Our repair algorithm for iatrogenic dissection starts with prompt recognition and evaluation of its origin and extent using transesophageal echocardiography. A surgeon who specializes in aortic surgery is often notified for intraoperative consultation. In catheter-based settings, angiographic diagnosis is generally made during the index procedure, and the patient is monitored for symptoms or signs of coronary malperfusion or root rupture. On occasion, iatrogenic dissections are noted only on follow-up computed tomography imaging.

When feasible, our repair strategy for standard DeBakey type I dissections involves a zone 2 arch repair as this facilitates future completion endografting.^8^ Iatrogenic dissections, however, represent a different physiology, given known location of the primary tear, limited time frame for formation of distal reentry tears, need to complete the index operation, and higher mortality than with standard dissections.^1^^,^^2^ Therefore, we elect to perform an aggressive hemiarch repair in most cases.^9^ Once the dissection is identified, percutaneous groin access may be obtained, but central true lumen cannulation is often possible by Seldinger technique with transesophageal echocardiography guidance. If the dissection is believed to be limited to a short segment of the otherwise normal ascending aorta, a high cross-clamp and closed distal technique may be used, but these situations are exceedingly rare. Total arch repairs are used in retrograde type A dissections, in our experience uniformly secondary to thoracic endovascular aortic repair (TEVAR). Should the root be deemed beyond standard “neomedia” repair, a complete root replacement is performed with or without valve-sparing techniques, depending on the quality of the valve, comorbidities of the patient, and index operation to be performed. Assessment of coronary malperfusion is also performed as previously described and may involve addition of coronary bypass grafts if indicated.^10^

## Results

During the study period, there were 1021 patients who were treated for type A dissections at our center, of whom 36 (3.5%) were confirmed to have iatrogenic causes. Of these, 23 (63.9%) were related to open surgical procedures (surgical incidence 0.1%, 5 (13.9%) to percutaneous coronary interventions, 5 (13.9%) to TEVAR, and 3 (8.3%) to other endovascular procedures (Figure).

**Figure:**
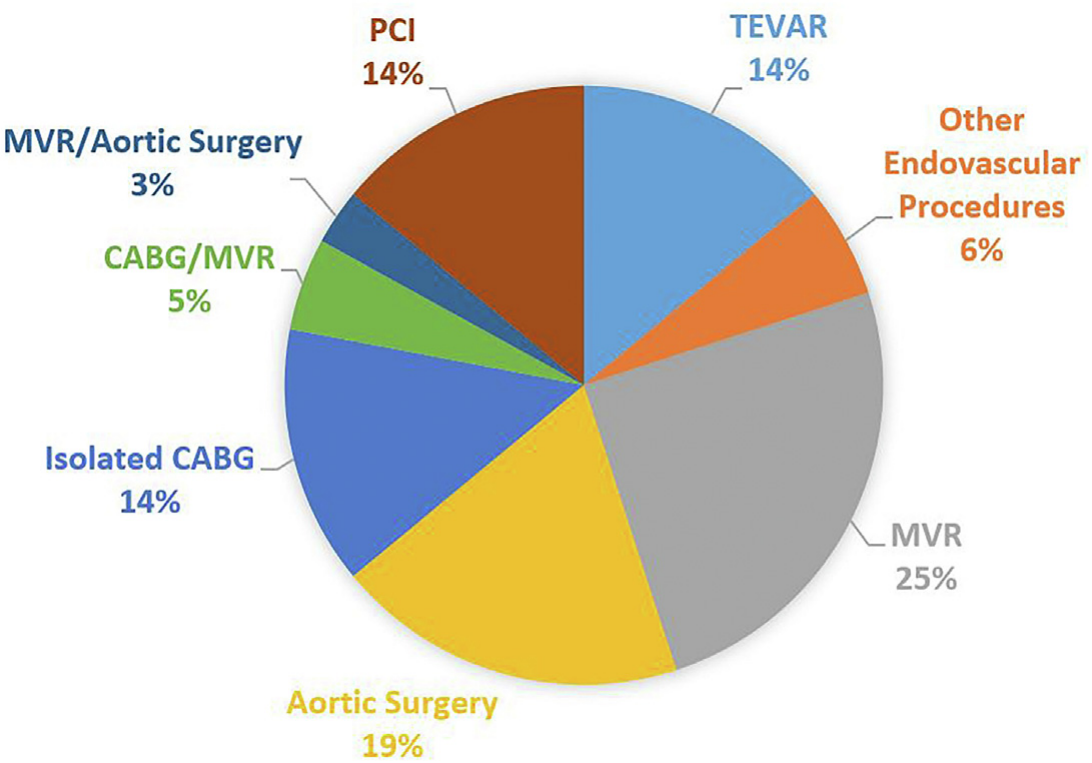
Distribution of causes of iatrogenic dissection. (CABG, coronary artery bypass grafting; MVR, mitral valve replacement; PCI, percutaneous coronary intervention; TEVAR, thoracic endovascular aortic repair.)

Baseline demographic characteristics of the patient cohort are detailed in the Supplemental Table; presenting features are noted in Table 1. Notably, most patients had dilated ascending aortas at baseline, with average size ∼4 cm. Dissection causes were varied; most often, dissections were related to cannulation site (9 [25%]), whereas 4 (11.1%) patients had dissections directly related to cross-clamping. In half of cases, dissections extended into the descending aorta; the remainder were limited to the ascending aorta.

**Table 1:** Presenting Dissection Features

Variables	Total No. (%) or Median (IQR)
Presentation	
Aortic insufficiency	7 (19.4)
Cardiogenic shock	5 (13.9)
Coronary malperfusion	10 (27.8)
Tamponade	2 (5.6)
Outside hospital transfer	4 (11.1)
Ascending aortic size, mm	39.5 (36-46)
Procedure type	
PCI	5 (13.9)
TEVAR	5 (13.9)
Open surgical	23 (63.9)
Other endovascular	3 (8.3)
Arterial cannulation strategy	
Central	25 (69.4)
Peripheral	11 (30.6)
Axillary	3 (8.3)
Femoral	8 (22.2)
Dissection cause	
Cross-clamp	4 (11.1)
Cannulation/decannulation	9 (25.0)
Catheter	8 (22.2)
Retrograde from TEVAR	5 (13.9)
Aortic retraction	2 (5.6)
Unknown/other	8 (22.2)
DeBakey classification	
Type I	16 (44.4)
Type II	16 (44.4)
Unknown	4 (11.1)

IQR, interquartile range; PCI, percutaneous coronary intervention; TEVAR, thoracic endovascular aortic repair.

Most of these patients underwent ascending aortic and hemiarch repairs under hypothermic circulatory arrest (28/36 [77.8%]), whereas those due to TEVAR typically required total arch repair. Repair strategies are further detailed in Table 2. One patient who presented with a dissection after an abdominal endovascular repair had evidence of an unstable, ruptured arch and required a total arch repair. Another patient who had a retrograde dissection after TEVAR had a classic bovine arch that thus could be repaired by a hemiarch technique while still sewing to stent graft material distally. Dissections requiring only ascending repair were noted in 2 cases. Root replacement was used in 6 (16.6%) patients.

**Table 2:** Operative Features

Variables	Total No. (%) or Median (IQR)
Cardiopulmonary bypass time, min	263 (207-301)
Hypothermic circulatory arrest time, min	26 (20-33)
Cross-clamp time, min	155 (119-190)
Repair procedure performed	
Ascending, hemiarch, felt root repair with AV resuspension	24 (66.7)
Ascending, total arch, felt root repair with AV resuspension	5 (13.9)
Ascending, felt root repair with AV resuspension	1 (2.8)
Bio-Bentall, ascending, hemiarch	4 (11.1)
Ascending, freestyle root	1 (2.8)
Ascending, hemiarch, freestyle root	1 (2.8)

AV, aortic valve; IQR, interquartile range.

Postoperative outcomes are detailed in Table 3. After initial surgical intervention, 16.7% (6/36) of patients required chest reexploration for bleeding, and postoperative strokes were noted in 3 (8.3%) patients. Among all patients, in-hospital mortality was 36.1% (13/36). Cause of death was most often cardiac, usually irrecoverable cardiac injury despite adjuvant mechanical support. Those who survived to discharge had low subsequent mortality, with no differences between endovascular and surgical causes (Supplemental Figure; *P* = .797). Median follow-up time was 3.1 years. After discharge, 11.1% (4/36) needed redo aortic surgery. These included 1 patient who underwent transcatheter aortic valve replacement for aortic stenosis at 6 years after discharge, 2 patients who required redo TEVAR for type IA endoleaks at 116 and 226 days after initial repair, and 1 patient who underwent residual type B dissection repair for enlarging diameter with TEVAR at 164 days postoperatively.

**Table 3:** Postoperative Outcomes

Variables	Total No. (%)
Reexploration for bleeding	6 (16.7)
Postoperative stroke	3 (8.3)
Required mechanical support	3 (8.3)
30-day mortality	13 (36.1)
In-hospital mortality	13 (36.1)
Redo aortic surgery	4 (11.1)
TAVR	1 (2.8)
TEVAR	3 (8.3)
Cause of death	
Cardiac	5 (13.9)
Sepsis	1 (2.8)
Respiratory	1 (2.8)
Stroke	1 (2.8)
Multisystem organ failure	1 (2.8)
Other	7 (19.4)

TAVR, transcatheter aortic valve replacement; TEVAR, thoracic endovascular aortic repair.

## Comment

Our institution’s approach for many acute type A dissections involves a more complete arch repair using a zone 2 arch strategy with subsequent endovascular repair with or without an interval revascularization procedure.^8^ At our center, this technique is achieved with similar circulatory arrest times to a standard hemiarch with appropriate dissection and preparation and also facilitates antegrade cerebral perfusion through axillary/direct innominate artery cannulation.^8^, ^9^, ^10^ However, when faced with iatrogenic aortic dissections, our strategy changes for a variety of reasons. Often, dissections are noted after cross-clamp removal or on attempted aortic decannulation, and repair warrants an additional bypass run. If the dissection is noted early in the case, the index procedure must still be performed after aortic repair, and in catheter-related dissections, the patient is often a nonideal surgical candidate. Thus, a repair strategy that offers as little additional time as possible should be employed. Furthermore, as these dissections are often focal in nature and occur secondary to iatrogenic manipulations as opposed to progression of aortopathy, a more limited repair strategy may be employed with infrequent need for remote aortic re-repair. Despite this, we think that an open distal technique is required in most cases and allows distal aortic reconstruction with felt neomedia and complete ascending replacement, limiting risk for need for redo repair.

In-hospital mortality in this series was 36.1%, within previously described reports and series. The largest series of iatrogenic dissections by Williams and coworkers^1^ demonstrated overall incidence at 0.06%, with high mortality (48.1%) and rates of chest reexploration and stroke (21% and 9%, respectively). In more contemporary series, mortality appears to be improving with more prompt recognition and treatment strategies. Rylski and colleagues^4^ reviewed the German Registry for Acute Aortic Dissection and noted an excellent 30-day mortality of only 16%. However, only 38% of their cohort received hemiarch replacement, and follow-up of these patients for remote aortic complications would be of great interest.

Notably, a disproportionate number of patients in this study had aneurysmal aortas at baseline, with an average aortic diameter of 4.0 cm. A similar finding was noted in the Columbia group’s reported experience, in which one-third of patients had an aneurysmal ascending aorta.^3^ Dissection causes were varied in our experience, the most common sources including cannulation sites or cross-clamp injury. However, dissections can occur from more unassuming sources, such as direct aortic manipulation and even endoballoon in 1 case during a minimally invasive mitral repair, emphasizing the need for care at every step when manipulating the aorta and a higher threshold for suspicion in baseline dilated aortas.

Dissections from catheter-based procedures represented about half of the cases in this cohort and a significant volume of our iatrogenic dissections. Nuñez-Gil and colleagues^7^ described 74 patients with catheter-related iatrogenic dissections, most of whom were managed with percutaneous coronary intervention of the dissection site and many others only conservatively. In their series, only 2 (2.7%) patients died of cardiogenic shock, and at a median follow-up of 51 months, no dissection-related complications were noted. Despite Dunning III dissections in 15 patients, only 3 were taken for cardiac surgery; the resolution of these dissections without surgical intervention is remarkable, considering that a multitude of these patients instead received percutaneous coronary intervention and were prescribed dual antiplatelet therapy. The decision to operate on catheter-based dissection thus must weigh the extent of dissection, frailty and wishes of the patient, and presence of complicating malperfusion. In this series, we describe 7 patients with extensive dissections who did proceed to surgery and overall a similar mortality to those with surgical iatrogenic causes. Among patients with retrograde dissections after TEVAR, total arch repair was thought to be necessary in most of these. These cases occurred during the early part of our experience with first-generation devices with uncovered metal stent proximal portions and higher radial strain. This complication has been greatly reduced with newer generation devices, and we have had no cases in the last 3 years.

In summary, we report a series of 36 patients undergoing surgical repair for iatrogenic ascending aortic dissections. We advocate for a uniform approach for most cases to include an open distal (hemiarch) technique. Mortality of patients with catheter-based or surgical iatrogenic causes was similar, and freedom from repeated aortic interventions for those who survived was excellent. Diligence must be paid to all aspects of aortic manipulation, especially in those patients with dilated aortas at baseline.
