# Supplementary material for: Iatrogenic Type A Dissection: Case Series and Surgical Management From a High-Volume Aortic Center
Source: Ann Thorac Surg Short Rep. 2023 Feb 27;1(2):272–6. doi: 10.1016/j.atssr.2023.02.011 (PMC11708600; doi:10.1016/j.atssr.2023.02.011)

**Supplemental Table 1: Preoperative Demographics**

| Variables | Total Number (%) or Median (IQR) |
| --- | --- |
| Average Age | 69 (61-77) |
| Male | 15 (41.7%) |
| Race |  |
| White | 29 (80.6%) |
| Black | 7 (19.4%) |
| Smoker | 4 (11.1%) |
| Hypertension | 26 (72.2%) |
| Diabetes | 4 (11.1%) |
| Chronic Lung Disease | 10 (27.8%) |
| Renal Failure | 2 (5.6%) |
| Bicuspid Aortic Valve | 1 (2.8%) |
| Pre-op CVA | 6 (16.7%) |
| Previous Cardiac Intervention | 19 (52.8%) |
| Preoperative Ejection Fraction | 49.4 (13.1) |

**Supplemental Figure 1: Patient Survival Curves by Etiology of Iatrogenic Dissection.** Log-rank p=0.797.


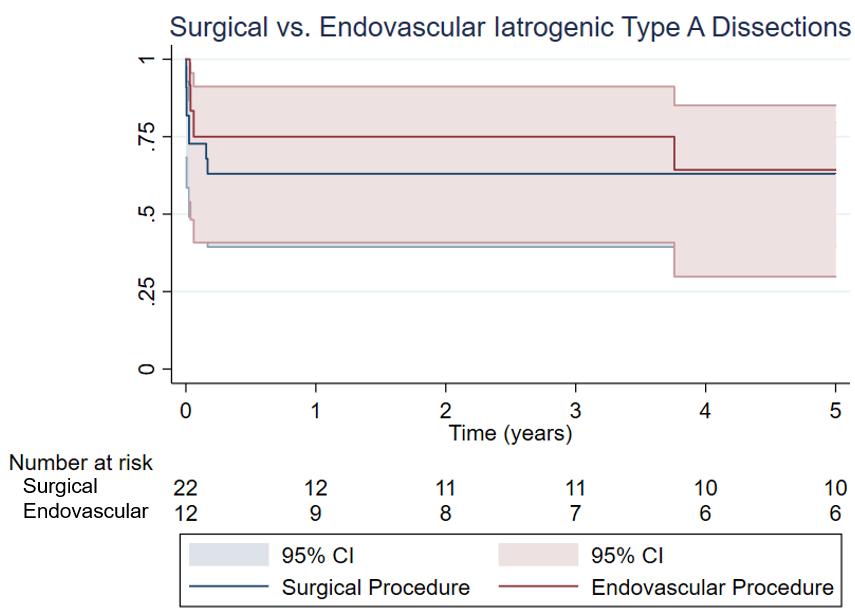

Supplement: Supplementary Material [file mmc1.docx]
